# Pre-decision regret before transition of dependents with severe dementia to long-term care

**DOI:** 10.1177/09697330211015339

**Published:** 2021-09-28

**Authors:** Ingrid Hanssen, Flora M Mkhonto, Hilde Øieren, Malmsey LM Sengane, Anne Lene Sørensen, Phuong Thai Minh Tran

**Affiliations:** 1 Lovisenberg Diaconal University College, Norway; 2 Sefako Makgatha Health Science University, South Africa; 3 VID Specialized University, Norway; 4 Sefako Makgatha Health Science University, South Africa; 5 Lovisenberg Diaconal University College, Norway; * Five authors are equally involved in the data collection, data analysis and authorship and are therefore all designated as second authors.

**Keywords:** Dementia, obligation, pre-decision regret, shame, stigma

## Abstract

**Background:**

To place a dependent with severe dementia in a nursing home is a painful and difficult decision to make. In collectivistic oriented societies or families, children tend to be socialised to care for ageing parents and to experience guilt and shame if they violate this principle. Leaving the care to professional caregivers does not conform with the cultural expectations of many ethnic groups and becomes a sign of the family’s moral failure.

**Research design:**

Qualitative design with individual in-depth interviews with nurses, family members and dementia care coordinators in Norway, Montenegro, Serbia and South Africa. Braun and Clarke’s six analytic phases were used.

**Ethical considerations:**

The project was approved by the Regional Committee for Research, South-Eastern Norway; the Norwegian Centre for Research Data; the Ethics Committee; University of Limpopo, MEDUNSA Campus, South Africa; and by the local heads of the respective nursing homes or home care services. Interviewees were informed orally and in writing and signed an informed consent form.

**Findings:**

Healthcare professionals tend to be contacted only when the situation becomes unmanageable. Interviewees talked about feelings of obligation, shame and stigma in their societies regarding dementia, particularly in connection with institutionalisation of family members. Many lacked support during the decision-making process and were in a squeeze between their own needs and the patients’ need of professional care, and the feeling of duty and worry about being stigmatised by their surroundings. This conflict may be a source of pre-decision regret.

**Conclusion:**

Family caregivers need help to understand the behaviours of persons with dementia and how to access the formal and informal services available. Thus, they may provide effective support to patients and family carers alike. Supportive interventions for caregivers need to be tailored to meet the individual needs of both the caregiver and the persons with dementia.

## Introduction

Placing a family member in a nursing home is for many family carers one of the most painful and morally difficult decisions to make,^1^ and it may be an intensely emotional experience.^2^ Several studies on the determinants of nursing home placement have been published.^1,3–10^ These are all focused on the perceptions and reactions of members of specific cultural groups. Other researchers focus on the physical, emotional, psychological and social burdens of being caregivers,^11–13^ or on familism or collectivism and the shame and stigma of not being able to care for the elderly family dependent on oneself.^1,11,14–19^

In this article, which is part of a larger study (Tables 1 and 2), we wish to investigate how family carers may experience making the choices of having their dependents with dementia admitted to a long-term care facility. We have found little research on this across collectivistic cultures as done in our study. Filling this knowledge gap is important as healthcarers need to understand what families across collective cultures go through in the process of making such a decision, and thus enable them to offer the support they need.

The research question is: What are the experiences of collectivistic oriented family members across cultures concerning choosing admission for a dependent with severe dementia to a long-term care facility?

It is estimated that 50 million people worldwide suffer from dementia in all its forms, and there are nearly 10 million new cases every year.^20^ This number may possibly reach 152 million in 2050.^20^ Dementia is a chronic neurodegenerative disease mainly associated with ageing.^21^ Patients with severe dementia will gradually develop behaviours perceived as deviant in most – if not in all – societies because of abnormal cognition, perception, mood or behaviour. These ‘include agitation, depression, apathy, repetitive questioning, psychosis, aggression, sleep problems, wandering, and a variety of socially inappropriate behaviors’ (p. 1).^22^ These are universal challenges which strongly impact family caregivers’ physical and mental health and well-being. Caring for persons with severe dementia may incur anxiety, emotional stress, poor sleep, exhaustion, depression and also sadness because their loved one is no longer the person they previously knew.^3,23,24^

### The decision process

Hirschfeld^25,26^ identified three factors, namely, capacity, tension and mutuality, which contribute to motivate placement of the family member with dementia in long-term care. The steeper the decline of the patient’s condition, the less the caregiver’s capacity to care. Tension refers to a combination of the number of unmet caring needs and their ascribed importance. Mutuality refers to the caregivers’ desire to find gratification in their relationship with the patient.

These factors may help understand the decision process, which can be parsed into three phases:

(1) The pre-decision phase, when alternatives are considered before a choice is made; (2) the period after the decision is made, but before the main outcome is disclosed, and (3) the post-outcome period, when decision makers are exposed to the full consequences of their choice. (p. 268)^27^

The third stage is not discussed in this article. Kwon and Tae^1^ have pragmatically named the two first decision process phases as (a) ‘realising a dead end’ and (b) ‘accepting the inevitable and reorienting to changes’.

The decision-making process is both cognitive and emotional.^24,28^ In collectivistic oriented societies or families, a central aspect of the pre-decision phase is that children are socialised to care for ageing parents and to experience guilt and shame if they violate this principle.^11,14,29^ In Korea, China, Japan and other East Asian countries, filial piety based on Confucianism is an important cultural norm, which emphasises the responsibility to care for aged parents.^1,7^ In Turkey and Morocco, family caregivers reported that admitting dependents to a nursing home is not done in their community.^30^ If a decision is made by the family to institutionalise the person with dementia, this may be condemned by the community. Thus, to have family members admitted to a care facility does not conform with the cultural expectations of many collective ethnic groups and becomes a sign of the family’s moral failure.^14,16,31^ This feeling of moral failure leads to shame and is accompanied by social stigma. Conditions of the mind, such as dementia, are particularly subject to stigma.^19^ Although shame is connected to a person’s internalised moral standards, Tangney et al.^32^ claim that it is a ‘public’ emotion as it is evoked by public exposure and disapproval.

Shame and stigma are among the aspects the family takes into account when making the decision to seek professional care for their dependent with dementia. When deciding what to do, the family caregivers will ruminate on their current situation and through this bolster what seems the most promising alternative. If this includes the institutionalising of the family member with dementia, its negative aspects were de-emphasised.^33^ The ‘promising alternative’ needs to ‘emerge as superior to’ the alternative(s) (p. 551).^33^ Although the caregiver(s) may feel ‘hope,…, perhaps accompanied by relief of having made up one’s mind’ in the pre-outcome interval (phase 2), there will also be uncertainties regarding the future and they may experience ‘negative prospective emotions, like anxieties, worry and fear’ (p. 268).^27^

## Method

This article is part of the research project, ‘Good dementia care in a multicultural society’. A qualitative design with individual in-depth interviews was chosen to acquire insights into the interviewees’ subjective experiences, attitudes and thoughts.^34–37^Qualitative research is well suited to study people’s reactions to moral dilemmas.^35^ The research question for the overall study was: What constitutes good care for patients with dementia according to nursing staff and patients’ family?

The starting point for this study was interviews with immigrant nurses caring for Norwegian patients with dementia in a care facility in Oslo (2010), based on experiences that communication problems and cultural differences might influence patient care. The study was gradually expanded to interviews of nurses and family members of Saami^i^ patients with dementia in a care facility in northern Norway (2010) and of nurses and family members to patients with dementia in care facilities in Risan, Montenegro and Aleksinac, Serbia (2010), and Tshwane, South Africa (2012).

### Participants

The goal was to interview 10 immigrant nurses in Oslo and 10 nurses and 10 family members in the other five locations. In Tshwane, 10 nurses and 10 family members at four different facilities were invited and agreed to be interviewed. We did not want to turn down any of these facilities/interviews and interviewed them all. Finally, as there are so far few immigrant patients in long-term facilities in Norway, five family members and three ‘dementia coordinators’ of home care in Oslo were interviewed (the Oslo-2 study, 2020). Thus, a total of 60 nurses and 42 family members were interviewed (Table 1).

**Table 1.** Overview of the total study. The interviews included in the current part-study is marked in gray.

Place/part-study	Number of Geriatric institutions	Nurses	Family members	Number of interviews containing the theme ‘pre-decision regret’		Interviewer(s)/background
				Nurses/dementia care coordinators	Family members	
Saami town,^1^ Norway	1	9	8	0	0	I.H./Norwegian
Oslo 1, Norway	1	6	0	1	–	I.H./Norwegian
Montenegro	1	11	7	9	2	A.L.S., I.H./Norwegian
Serbia	1	12	3	6	2	A.L.S, I.H./Norwegian
Tshwane, South Africa	4	19	19	2	7	F.M.M./Xitsonga M.L.M.S./Tshivenda H.Ø., I.H./Norwegian Y.H./Afrikaans
Oslo 2, Norway	Home care districts 4	Dementia care coordinators 3	5	3	2	P.T.M.T./Vietnamese and Norwegian I.H./Norwegian
Total	12	57	42	21	11	

### Inclusion criteria

Nurses experienced in caring for patients with dementia and family members above 18 years of age with dependents admitted to a long-term care facility. The heads of the respective geriatric institutions chose what interviewees to invite as participants based on their knowledge of the patients and their family members.

The interview guide question relating to this article was: What constitutes good care for the patients with dementia you care for/your dependent in this care facility? The nurses were additionally asked: If your mother/father develop dementia, will you have her or him admitted to a long-term care facility like the one you are working in?

### Data collection

The Oslo-1 and one of the Oslo-2 interviewees had Asian backgrounds. While all but two of the Tshwane family interviewees were Afrikaaners,^ii^ the majority of the nurses had various African cultural backgrounds. The interviews took form of an electronically recorded talk where the respondents were encouraged to share their thoughts and recount their experiences. Follow-up questions and the ‘mirroring’ of statements were used to develop, clarify and verify statements. In South Africa, the Balkans and Oslo-2 workshops were held before the interviews commenced to create a communal understanding of the research method. Workshops were also held before and after each day of interviewing to synchronise the method used and share experiences and findings. All the researchers are nurses. Two of the South African team members are experienced researchers and I.H. is experienced in intercultural qualitative research.

### Data analysis

In South Africa and the Balkans, workshops were held after the conclusion of all these part-studies’ interviews to discuss what main themes were found. I.H. transcribed all the interviews verbatim. Analytic rigour was obtained by being six analysts.

We used Braun and Clarke’s^38^ six analytic phases for thematic analysis. (1) The authors familiarised themselves with the interview data through reading and re-reading the interview texts actively searching for meanings and patterns. Reflective thoughts were documented along the way. A first analysis was done to find what main themes were identified in the data (Table 2). ‘Pre-decision’ regret became one such theme although none of the interviewees actually used this term.

**Table 2.** Main themes gleaned from the study’s interviews.

Main themes	Part-studies involved	Published article
Music as means for remembrance among persons with dementia	The Saami	√
The influence of cultural background in intercultural dementia care	The Saami	√
Traditional food in dementia care	The Saami, Tshwane	√
Language and cultural diversity in intercultural dementia care	The Saami, Oslo-1, Tshwane	√
Superstitions concerning people with dementia	Tshwane	√
Individualism, collectivism and care choices	Oslo-1 & Oslo-2, The Balkans, Tshwane	√
Pre-decision regret before transition to long-term care	Oslo-1 & Oslo-2, The Balkans, Tshwane	Present study

The interviews containing these main themes were then re-analysed theme by theme (Table 2). (2) During each re-analysis, interesting features were coded (phase 2). We kept revisiting the interviews focused on specific characteristics of the data (Table 3). During phases 2 and 3, the collation of potential sub-themes,^38^ our thoughts and ideas evolved from engaging with the data. Thus, the creation of themes was data-driven and inductively generated.^37^

**Table 3.** Re-analysis of pre-decision regret as main theme by sub-themes, analytic phases 2–4.

Main theme based on analysis of all the interviews:	No. of interviews analysed	Phase 2	Phase 3	Phase 4
Pre-decision regret	32	• Wanting to care for dependent who took care of them growing up • Expected to care for the person • Professional care culturally inacceptable • No longer able to cope	• Obligation • Failure • Stigma • Professional care only viable option	• Obligation • Shame • Stigma • Pre-decision regret

(4) We reviewed the main theme, which was the focus for re-analysis – here: ‘pre-decision’ (Table 1, 32 interviews) – and its sub-themes (Table 3) and discussed whether these reflected the meanings evident in the re-analysed dataset as a whole.^38^ (5) We discussed whether the sub-themes developed described the theme’s content. (6) The first author’s preliminary text was discussed and developed further collaboratively.

Thus, the analytic process was repeated for each main theme. Analytic credibility is obtained through quotations with interviewee’s own description of thoughts and experiences.^37^ This and thick descriptions furthermore strengthen confirmability and trustworthiness as they show that the findings are based on our interviewees’ responses and not on potential bias.^37^

### Ethical considerations

The project was approved by the Regional Committee for Research, South-Eastern Norway; the Norwegian Centre for Research Data; the Ethics Committee, University of Limpopo, MEDUNSA Campus, South Africa; and by the local heads of the respective nursing homes. All interviewees were informed orally and in writing that participation was confidential and voluntary, and that they were free to withdraw from the project at any time. They all signed an informed consent form. To ensure anonymity, the Montenegrin and the Serbian data are co-presented as Balkan data and the Oslo-1 and Oslo-2 data are co-presented as Oslo data. The recorded interviews were deleted after transcription. Transcriptions are stored according to ethical research guidelines.^39^

### Critical remarks

One of the Oslo-2 interviews was conducted in Vietnamese and the rest of the Oslo interviews in Norwegian. The Balkan interviews were assisted by a native Serbian and Norwegian speaker. Most of the Tshwane interviews were conducted in English, a few in Afrikaans and Setswana. During some of the interviews, the talk would have run easier and the content been richer if the interviewees had spoken a language with which they were more comfortable. The offer to be interviewed in their respective home language should have been communicated in a way in which the choice of language could not be perceived as a question of pride.

The researchers had no influence on which interviewees were invited as participants. Although this might have skewed the choice towards interviewees with a positive view of the respective facilities, criticism was vented in all locations. I.H. took part in the initial interviews all the co-researchers conducted, and in Tshwane, she rotated between the co-researchers to ensure similar interview approaches. The South African co-authors mastered all the languages used by the South African interviewees. With their variety of cultural backgrounds, the researchers represented both an insider and an outsider view during the interviewing as well as during the data analysis.

## Findings

Although there was some mention of stigma in connection with dementia, pre-decision regret, shame and obligation were not topics among the Saami interviewees. All these themes were found in the data from the other locations. Particularly in many of the Balkan and Tshwane interviews, ‘pre-decision regret’, although this particular term was never used, was a main topic.

The path for family carers to place a family member in a nursing home is organised into two main themes: (1) having to recognise inability to cope and (2) obligation, shame and stigma.

### Having to recognise inability to cope

Despite what is expected of them, caring for a dependent with severe dementia is exhausting. Independent of cultural background, all the family carers talked about the hardships of caring for their dependents at home. An Oslo family interviewee, for instance, related how her husband would shout and cry whenever she left the apartment. He would panic as soon as she was out of view thinking that she had left him and gone back to their home country. In the end, admitting him to a nursing home was the only solution.

Such hardships seemed to be the main reason why they decided to have their dependent institutionalised. If not, ‘it kills you, but very slowly. And if it doesn’t kill you, it will kill your family, it will kill your children’, as a Tshwane caregiver put it. What ‘kills’ families and forces them to make this decision, is the change in the behaviour of their dependent with dementia, such as forgetting to turn off the kitchen cooker, accusing family members of stealing, the endless repetitions and questions, having to organise every day according to the dependent’s needs and exhaustion from being on the alert and caring for the dependent around the clock. This situation made caregivers feel ‘imprisoned’ and ‘trapped’. Even so, many felt that one should endure ‘and does not discuss any different solution.…one is to nurse one’s parents at home, regardless’ (Balkan nurse).

When ‘they need to be around you all the time, that’s not always possible because obviously you sort of get claustrophobic. You feel as if *you* don’t have a life anymore. So, then you go on the guilt-trip again’ (Tshwane caregiver). The fact that family caregivers are unable to look after their ill family members 24/7 was a theme among several of the interviewees.

### Obligation, shame and stigma

The ethnic African nurses, although favouring family care, saw the importance of professional care/institutionalisation when a dependent’s dementia became severe. Only six of the Balkan nurses and one Oslo nurse said they would choose to place their parents in a nursing home if suffering from severe dementia. The rest of these nurse interviewees were more ambivalent about placing a family member in a care facility or rejected such a move outright:

It is my duty as daughter to nurse my parents.…I would probably have tried to nurse my parents as long as possible,…There have to be such institutions and there is a great need for them, but I think it is right to care for the family’s old [oneself]. (Balkan nurse)

Furthermore, caring for family members ‘is my task. My parents cared for me when I was a baby; they did everything for me. Now my mother is ill, and I threw her out’ (Oslo caregiver). These family caregivers had done everything they could to care for their dependents at home for as long as possible.

An Oslo dementia care coordinator held that in immigrant families ‘one feels that one wants to care for one’s parents oneself. And when one no longer is able to cope, one feels shame’. Both Balkan and Oslo interviewees talked about the shame and stigma in their societies regarding dementia, particularly in connection with institutionalisation of family members. A Balkan nurse explained that ‘a person is denounced if someone sends their family member to a nursing home. It is still shameful…I am not able to picture myself placing [my parent] in a nursing home’. Another Balkan nurse held that

It is very stigmatising. The attitude is that it is totally unacceptable that persons who have nurtured us and cared for us in our younger days and as adults, when the day comes that they need nurturing and nursing care, are being stowed away. That is perceived as turning one’s back on them.

This is despite many patients being left to their own devices while the families’ children are at school and the adults are at work.

An Oslo caregiver held that

in our culture we do not send our parents to nursing homes. We care for them ourselves. But here in Norway this is impossible.…As we live here in Norway [my compatriots] say that it is customary, but deep down inside they do not think it is the right thing to do.

Worry about family members’ and neighbours’ reactions was also important: ‘What I care about is what my closest family would say to me.…I would have had problems with my family’ (Balkan nurse). In the Balkans, we were told that some families had their dependent admitted to an institution in another part of the country to avoid stigma and blame from their neighbours.

Dementia care coordinators often found it difficult having professional care accepted in the immigrant population even when both the patient and the family caregivers were in dire need of assistance. In one case, a daughter had had to move to Norway to help her brother and his wife care for their father as they no longer were able to cope on their own and did not want professional help.

In South Africa, both availability of care facilities and economy were important factors regarding transition to long-term care. Most of the nursing homes are in the cities, which means that people living in the outskirts of towns may not access them. As the families often have to pay most of the fees themselves, this excludes the disadvantaged part of the population further unless patients are admitted pro bono. One daughter said that ‘I think there is a shortage in South Africa,….and if people can’t afford it, there is a bit of a problem. I think the State should contribute more,…I’ve never seen a black person [in this facility]’.

Lack of knowledge about the development of the disease may also make the decision concerning professional care even more difficult. One of the Oslo caregivers, for instance, had thought that she would be able to care for her mother if only she was given the financial resources from social services to rent a larger apartment. She was told her mother was too ill to be cared for at home. The daughter became very frustrated: ‘Why, when I had coped with her care for eight months?’ However, she gradually understood, ‘what they meant when they said that she would become worse and worse…that after a while I would not be able to [care for her]. And that is how it is. But back then I felt that they forced me to send my mother to an institution’. According to one of the Oslo dementia care coordinators, many family carers find it difficult to accept the offer of professional care even when in dire need of such assistance.

## Discussion

The discussion is focused on the decision process across cultures and the ensuing pre-decision regret when especially collectivistic oriented family caregivers turn to professional care in long-term nursing homes.

### Strong obligation, neglecting their own needs

We found that although a great challenge, our interviewees’ strong feeling of being obligated to care for their parents was a strong barrier to seek professional help and long-term care. This is often connected to a collectivistic world view.^8,11,14,30^

Spouses tend to feel that they have to neglect and/or adjust their own needs to prioritise the needs of the person with dementia^6,12^ as they have to observe and supervise him or her around the clock. Caregivers find it particularly difficult to manage wandering, psychotic behaviours^12^ and aggression.^11^ This was mirrored among our interviewees as many found themselves ‘trapped’ and ‘imprisoned’. These feelings may intensify as the tension between their dependent’s caring needs and their capacity to meet these needs increases.^25,26^ The problem is further exacerbated by societal changes^11^ and changes in family life and dynamics.^7–11,25,28^ Many caregivers find themselves in a squeeze between their need of respite from their care burden and the patients’ need of professional care, on the one hand, and the feeling of duty and worry about being stigmatised by their surroundings, on the other.^7,14,28,40^

According to Wang et al.^11^ ‘most spouse caregivers and the people with dementia are isolated at home and have limited contact with others’ (p. 1375). This is not least the case in areas where people with severe dementia are believed to be witches.^41^ Hence, caregivers may lack support during the decision-making process and be blamed for skirting their duties after the patient’s nursing home transition. Also, families in more individualistic societies tend to want to care for dependents with severe dementia at home as long as possible. However, these caregivers often receive more support from their surroundings as professional care tends to be more socially accepted.^4^

### Feelings of shame, stigma, failure and regret

Except among the Saami and Tshwane Afrikaaner interviewees, the stigma of dementia seemed to make families hesitate to seek professional help even when memory problems and other symptoms were severe, and help was needed. The Oslo and Balkan family interviewees clearly indicated that they still wanted to care for their parents themselves, and when the decision to seek professional care was unavoidable, they felt guilt, shame and pre-decision regret.^7,27^ Park et al.^7^ found that particularly older caregivers ‘expressed their concern for other people’s view and shame for their decisions’ (p. 351). To avoid blame, carers may try to keep the fact that a family member has dementia ‘in the family’ as exemplified by families sending dependents to institutions far from home to avoid ‘detection’.

**Figure 1.**
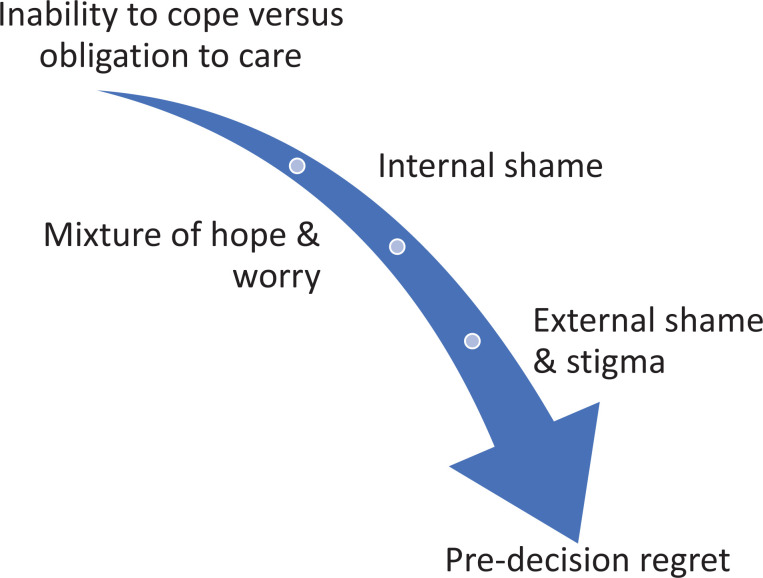
The pre-decision interval leading to pre-decision regret.

Although the ability to cope with the care within the family tends to be a question of honour and duty, many families gradually find this to be no viable solution.^7,24^ When family caregivers reach this point, they have passed through the pre-decision phases of Kirkebøen and Teigen^27^ during which alternatives are considered, and they have made their decision (Figure 1). They have realised that they have reached a ‘dead end’ and accepted that change is inevitable.^1^ To get to this point, they have gone through a variety of cognitive processes. While they may harbour prospective emotions of hope and relief, they may also experience worry, shame and guilt, even if they realise that they no longer are able to care for their ageing dependent.^29^ Their pre-decision regret may also include that they will miss the feeling of mutuality with their dependent and the gratification their relationship offers them.^25,26,42^ They may also feel that they, by admitting a family member to a care facility, have failed their obligation since to do so does not conform with the cultural expectations of many ethnic groups and becomes a sign of the family’s moral failure.^14,16,24,30^

The stigma of institutionalisation of dependents with dementia may have different culture-based reasons, although the shame and stigma related to caregivers not honouring their obligation seem to be the most common view. Being related and having received care from childhood from the persons who now need assistance themselves seem universally to create a special obligation.^43^ The conflict between the carers’ needs of respite from their care burden combined with a feeling of obligation may be an important source of regret. The experienced stress from making the decision and preparing for the dependent’s move may cause further regret, together with doubts concerning the moral appropriateness of the decision. Thus, regret may ‘be due to a fear of consequences,…or simply a renewed evaluation of the moral appropriateness of the…decision’ (p. 268).^27^

According to Arneson,^44^ shame stems from feeling negatively judged according to the dominant standard of a given society or group. This is reflected in the interviews with several of the Balkan and Oslo interviewees. Many of them were worried about ‘loss of face’. Public loss of face may be seen as public stigma, which refers to the reactions of lay persons towards a stigmatised individual or group.^14^

Goffman^31^ held that we are not aware of the society’s demands upon us ‘until an active question arises as to whether or not they will be fulfilled. We then realize that certain assumptions are being made about what is expected of us’ (p. 12). These expectations typically elicit a social process ‘characterized by exclusion, rejection, blame or devaluation’ (p. 441).^45^ To avoid this, carers may make a valiant effort ‘to balance their caregiving role with maintaining previously held relationships, even though this [may prove] difficult’ (p. 240)^18^ or even impossible. Even if the family carers have some knowledge about dementia, they may lack insight into how physical and mental symptoms are related to the development of the disease.^46,47^ There will, in most cases, come a time when seeking professional care is starting to loom in the horizon as unavoidable. At such a time, the carer realises having reached ‘a dead end’^1^ but is still considering what to do. Until the carer accepts ‘the inevitable and successfully reorganises her/his thoughts and feeling to changes’,^1^ this may be a period of worry and serious pre-decision regret.

## Conclusion

In this article, we have discussed the decision to have a dependent with severe dementia receive professional care in light of pre-decision regret, obligation, shame and stigma across collectivistic cultures. Buhr et al. hold that ‘[h]ealth care providers who directly raise the issue for discussion before caregivers reach a “breaking point” could ease the transition by offering these carers their guidance and support’ (p. 52).^12^ Family carers need to be made aware of the formal and informal services available and how to access these. Furthermore, all healthcare practitioners should be trained to help family carers to find confidence in their decision to have their dependent with severe dementia transferred to professional care. Being genuinely interested in what is important both to the patient and to the caregivers will enable healthcarers to help families put worries to rest and through collaboration find the best possible solutions for the patient.

Would this article’s findings have been similar if we had interviewed ethnic Norwegian family caregivers? This is a question which needs to be studied. However, our present findings may help explain why there still are few immigrant patients with dementia in Norwegian and in Western European nursing homes.

## Notes

i. The Saami is indigenous people of Norway.

ii. Afrikaaners are people of European heritage who speak Afrikaans, a Low Franconian West Germanic language descended from Dutch and spoken mainly in South Africa and Namibia (Ager, undated).
